# Emergence of fluoroquinolone resistance among drug resistant tuberculosis patients at a tertiary care facility in Karachi, Pakistan

**DOI:** 10.1186/s13104-017-2633-6

**Published:** 2017-07-25

**Authors:** Syed Mohammad Asad Zaidi, Abdul Haseeb, Shifa Salman Habib, Amyn Malik, Saira Khowaja, Nausheen SaifUllah, Nadeem Rizvi

**Affiliations:** 1 Interactive Research & Development, Suite 508, Ibrahim Trade Tower, Main Shahrah-e-Faisal, Karachi, Pakistan; 2 Department of Chest Medicine, Jinnah Post Graduate Medical Centre, Rafiqi H J Rd, Karachi, Pakistan

**Keywords:** Multi-drug resistant tuberculosis, Fluoroquinolone resistance, Pakistan

## Abstract

**Background:**

Pakistan is classified as one of the high multi-drug resistant tuberculosis (MDR-TB) burden countries. A poorly regulated private sector, over-prescription of antibiotics and self-medication has led to augmented rates of drug-resistance in the country. Pakistan’s first national anti-tuberculosis drug resistance survey identified high prevalence of fluoroquinolone resistance among MDR-TB patients. Further institutional evidence of fluoroquinolone drug-resistance can support re-evaluation of treatment regimens as well as invigorate efforts to control antibiotic resistance in the country.

**Findings:**

In this study, data for drug-susceptibility testing (DST) was retrospectively analyzed for a total of 133 patients receiving MDR-TB treatment at the Chest Department of Jinnah Postgraduate Medical Center, Karachi, Pakistan. Frequency analyses for resistance patterns was carried out and association of fluoroquinolone (ofloxacin) resistance with demographics and past TB treatment category were assessed. Within first-line drugs, resistance to isoniazid was detected in 97.7% of cases, followed by rifampicin (96.9%), pyrazinamide (86.4%), ethambutol (69.2%) and streptomycin (64.6%). Within second-line drugs, ofloxacin resistance was detected in 34.6% of cases. Resistance to ethionamide and amikacin was 2.3% and 1.6%, respectively. Combined resistance of oflaxacin and isoniazid was detected in 33.9% of cases. Age, gender and past TB treatment category were not significantly associated with resistance to ofloxacin.

**Conclusion:**

Fluoroquinolone resistance was observed in an alarmingly high proportion of MDR-TB cases. Our results suggest caution in their use for empirical management of MDR-TB cases and recommended treatment regimens for MDR-TB may require re-evaluation. Greater engagement of private providers and stringent pharmacy regulations are urgently required.

## Findings

### Background

Globally, emerging drug resistance poses a serious threat to the control of tuberculosis (TB) [1]. In 2012, the World Health Organization (WHO) estimated 450,000 (range 300,000–600,000) new cases of multidrug-resistant TB (MDR-TB) in the world; 3.6% of newly diagnosed TB cases, causing an estimated 170,000 (range 102,000–142,000) deaths [2]. Pakistan contributes to 69% of the MDR-TB burden in the Eastern Mediterranean Region (EMRO), with an estimated 11,000–29,000 cases occurring in 2012 [2]. Over the past few years the country has also reported an increasing trend of Extensively Drug Resistance disease (XDR-TB) that includes resistance to fluoroquinolones and at least one other second-line injectable, in addition to MDR-TB [3].

Fluoroquinolone resistance is of particular public health importance as it is a widely prescribed antibiotic for treatment of common respiratory tract infections. There is a rampant and poorly regulated private healthcare sector in Pakistan, where over-prescription of fluoroquinolones by general practitioners, over-the-counter sales at private pharmacies and self-medication by patients occur commonly [4]. Recently published findings from the first national anti-tuberculosis drug resistance survey of Pakistan identified fluoroquinolone resistance of 26.6% (95% CI 18.0–36.7) among a total sample of 96 MDR-TB cases [5]. Evidence suggests that occurrence of fluoroquinolone resistance is on the rise, with a study reporting increase in its prevalence from 17.4% in 2005, to 53.9% in 2014 among MDR-TB patients [6].

Fluoroquinolones are the mainstay of treatment of MDR-TB and resistance has been associated with poor treatment outcomes for MDR-TB patients [6]. Rising fluoroquinolone resistance is, therefore, of serious concern for the management of MDR-TB patients, particularly at a time when Pakistan is scaling-up its Programmatic Management of Drug-resistant TB (PMDT) program [7]. Scale-up of Xpert MTB/RIF testing in the country has significantly facilitated diagnosis of MDR-TB since resistance to rifampicin can be identified at the time of diagnosis [8]. However, patients initially require empirical treatment as drug-susceptibility testing (DST) results to fluoroquinolones and other second-line drugs require 6–8 weeks by conventional methods. A lack of clinical response to medication can be alarming for both patients and providers [9]. In the setting of increasing resistance to fluoroquinolones, current combinations of empirical therapy used by MDR-TB programs may require revaluation. Further institutional evidence of fluoroquinolone drug-resistance among MDR-TB patients can support such recommendations as well as boost efforts to control antibiotic resistance in the country. This study describes the drug-resistance patterns of microbial isolates from MDR-TB suspects from a tertiary-care facility in Karachi, Pakistan from 2009 to 2012.

### Methods

We analyzed de-identified, retrospective data of patients receiving MDR-TB treatment at Jinnah Postgraduate Medical Center, the largest public-sector hospital in Pakistan’s southern Sind province, between 2009 and 2012. All patients enrolled on MDR-TB program had a history of TB treatment and were included in the analysis using convenience sampling. Sputum was obtained from the patients prior to MDR-TB treatment initiation and DST of *Mycobacterium tuberculosis* isolates was carried out at the Aga Khan University clinical laboratory, a technical partnering facility for the National TB Program (NTP). Demographic and clinical histories of the patients were obtained from the hospital records. Treatment history was categorized as prior history of Category I TB treatment (four-drug anti-TB treatment regimen) and Category II (four-drug anti-TB treatment regimen + streptomycin). Frequency analyses for resistance patterns and demographic variables were carried out. The association of ofloxacin (a second-generation fluoroquinolone) resistance with age was investigated using Student’s t test. Chi squared tests were conducted to investigate the association of ofloxacin resistance with gender and treatment history. Statistical analyses were carried out using Stata/IC 12.0 (Stata Corp, College Station, TX, USA).

### Results

A total of 133 patients receiving MDR-TB treatment were included in the study. Median age of the patients was 16 (IQR 10–26) and 51.1% were male (Table 1).

**Table 1:** Demographic characteristics of MDR-TB patients at Jinnah Postgraduate Medical Center, Karachi, Pakistan (2009–2012)

	n	(%)
Age (years)		
9–14	10	(8)
15–29	71	(54)
30–44	33	(25)
45<	19	(14)
Gender		
Male	68	(51.1)
Female	65	(48.9)
Total	133	

Within first-line drugs, resistance to isoniazid was reported in 97.7% of cases followed by rifampicin (96.9%), pyrazinamide (86.4%), ethambutol (69.2%) and streptomycin (64.6%) (Table 2).

**Table 2:** Resistance to first and second-line anti-tuberculous medication among MDR-TB patients at Jinnah Postgraduate Medical Center, Karachi, Pakistan (2009–2012)

	Number of cases with resistance	
	n	(%)
Any resistance (first line drugs)		
Isoniazid (H)	130	(97.7)
Rifampicin (R)	129	(96.9)
Pyrazinamide (Z)	115	(86.4)
Ethambutol (E)	92	(69.2)
Streptomycin (S)	86	(64.6)
Any resistance (second line drugs)		
Kanamycin (Km)	1	(0.8)
Amikacin (Am)	2	(1.5)
Capreomycin (Cm)	2	(1.5)
Ofloxacin (Ofx)	49	(34.6)
Ethionamide (Eto)	3	(2.3)
Cycloserine (Cs)	1	(0.8)
Combined resistance of H with other drugs		
H + Z	114	(85.7)
H + E	91	(68.4)
H + S	86	(64.7)
H + Z + E	90	(67.7)
H + Z + E + S	67	(50.4)
H + Ofx	45	(33.9)

Within second-line drugs, ofloxacin resistance was observed in 34.6% of cases. Resistance to ethionamide and amikacin was 2.3 and 1.6%, respectively (Table 2). Combined resistance to ofloxacin and isoniazid was reported in 33.9% of cases (Table 2). Ofloxacin resistance was more commonly observed in patients with history of Category I treatment (54.3%) compared to Category II (40.7%), however, the difference was not statistically significant (p value 0.26). Age (p value 0.92) and gender (p value 0.85) were not significantly associated with ofloxacin resistance. A total of 3 cases were found to have XDR-TB.

### Discussion

Our results support findings from previously reported studies and the national anti-TB drug resistance survey, highlighting growing resistance to fluoroquinolones in Pakistan. This is the first study describing institutional evidence from an MDR-TB treatment facility of the high prevalence of fluoroquinolone resistance among MDR-TB cases. Resistance was not associated with age, gender or history of TB treatment, suggesting that resistance may be widespread within this patient population. These findings raise concerns for the outcomes of MDR-TB patients and for empirical treatment, following rifampicin resistance identification through an Xpert MTB/RIF assay. Recommended treatment regimens for MDR-TB may therefore require revaluation for improved treatment outcomes and to curtail development of further drug-resistance.

Given the above scenario, several public health policy measures and programmatic interventions can be of benefit. Firstly, the use of antibiotics, particularly fluoroquinolones needs to be strongly regulated. This is likely to be a major challenge, given Pakistan’s large and fragmented private healthcare sector. A regulation restricting over-the-counter sale of antibiotics also needs to be introduced, the practical implementation of which could be difficult. Secondly, strategies to engage with the private-sector providers more comprehensively need to be explored. Recent initiatives proposed by the National TB Control program are encouraging [10]. Under novel public–private mix approaches, local networks and partnerships can be created where private providers receive medication through provincial TB control programs for patients and in turn, help to increase case-notification. Greater efforts should be in place to collaborate with general practitioners, especially with family practitioners operating in small clinics in urban areas. Trainings should be conducted on NTP guidelines for TB case-detection and treatment as well as on more judicious use of antibiotics for treatment of common infections, with a special emphasis on restricting use of fluoroquinolones. Medical associations, including those representing infectious disease specialists, pulmonologists as well as pharmacists need to be play a more active role in communicating this message to providers and to the public. Finally, MDR-TB programs will require greater efforts to improve case-holding and ensuring treatment completion for enrolled patients, as PMDT services expand in the country. Community-based solutions and behavioral economics approaches such as the use of food-baskets and other incentives for treatment compliance may be of benefit [11]. Early case-detection through scale-up of Xpert MTB/RIF testing may help improve treatment outcomes as well as limit transmission of resistant strains. This study was limited by the absence of DST results for third and fourth generation fluoroquinolones for assessing cross-resistance with ofloxacin of *M. tuberculosis* isolates. In addition, limited patient history was available, such as prior history of quinolone use, to further investigate risk factors and clinical correlates to fluoroquinolone resistance. We recommend further studies to evaluate MDR-TB treatment outcomes and their association with fluoroquinolone resistance among DR-TB patients.

### Conclusions

Fluoroquinolone resistance was observed in an alarmingly high proportion of MDR-TB cases. Our results suggest caution in their use for empirical management of MDR-TB cases (particularly second-generation) and therefore recommended treatment regimens for MDR-TB may require revaluation. Engaging private sector providers in TB case-detection and stringent pharmacy regulations are required to prevent increased resistance to fluoroquinolones in Pakistan.

## Abbreviations

TB: tuberculosis
MDR-TB: multi-drug resistant tuberculosis
EMRO: Eastern Mediterranean Region
XDR-TB: extensively drug resistant tuberculosis
PMDT: Programmatic Management of Drug-resistant
DST: drug Susceptibility testing
NTP: National Tuberculosis Control program
MTB/RIF: mycobacterium tuberculosis/rifampicin
